# A Comparative Analysis of Mucus Immunomodulatory Properties from Seven Marine Gastropods from the Mediterranean Sea

**DOI:** 10.3390/cells11152340

**Published:** 2022-07-29

**Authors:** Clementina Sansone, Cecilia Balestra, Luigi Pistelli, Angelo Del Mondo, David Osca, Christophe Brunet, Fabio Crocetta

**Affiliations:** 1 Department of Marine Biotechnology, Stazione Zoologica Anton Dohrn, Villa Comunale, I-80121 Napoli, Italy; cbalestra@ogs.it (C.B.); luigi.pistelli@szn.it (L.P.); angelo.delmondo@szn.it (A.D.M.); christophe.brunet@szn.it (C.B.); 2 Institute of Biomolecular Chemistry, National Council of Reasearch, Via Campi Flegrei 34, I-80078 Pozzuoli, Italy; 3 National Institute of Oceanography and Applied Geophysics—OGS, I-34100 Trieste, Italy; 4 Department of Integrative Marine Ecology, Stazione Zoologica Anton Dohrn, Villa Comunale, I-80121 Napoli, Italy; david.osca@ulpgc.es (D.O.); fabio.crocetta@szn.it (F.C.)

**Keywords:** immunomodulation, marine biotechnology, gastropods’ mucus, monocytes’ differentiation

## Abstract

The treatment of inflammatory and immune-related diseases due to dysfunctioning of the immune system necessitates modulation of the immune response through immunomodulatory compounds. Marine environments are considered as a new frontier for health benefit product implementations. Marine biodiversity is still a low explored resource, despite it is expected to represent an important platform for chemical bioactive compounds. Within the phylum Mollusca, gastropods are known to synthetize mucus, the latter presenting relevant bioactive properties, e.g., related to immunomodulant molecules able to activate the innate and acquired immune system. This study proposes a bioprospecting of the immunomodulant activity of mucus isolated from seven common gastropod species from the Gulf of Naples (Mediterranean Sea). Results showed that not all mucus displayed a significant cytotoxic activity on the two human cancer cell lines A549 and A2058. On the other hand, the mucus from *Bolinus brandaris* was strongly bioactive and was therefore thoroughly investigated at cellular, molecular, and protein levels on the human monocytes U937 line. It can conclusively induce monocyte differentiation in vitro and significantly stimulate natural immunity response.

## 1. Introduction

Immune system dysregulation is often related to inflammatory diseases and chronic illnesses such as obesity, diabetes, cancer, rheumatoid arthritis, and neurodegenerative and autoimmune diseases [1]. Chronic inflammation might be a problem for human health, being the major cause of immune-mediated diseases such as bowel disease or type 1 diabetes mellitus [1], which in turn have a high cost for society [2]. In 2011, this has been estimated in the USA as being about $100 billion per year. The discovery and development of new immunotherapeutic agents that reprogram and maintain/restore immune system homeostasis is therefore a medical and societal requirement. Oceans contain the world’s widest biodiversity in terms of animal and plants species [3]. The adaptive strategies against hostile and competitive oceanic environments, especially for benthic species, promote the greatest and most unique chemo-diversity along the marine biodiversity axis. This resource therefore constitutes an opportunity for human health-related biotechnology, as known since antiquity [4]. Indeed, a plethora of marine compounds show significant antiviral, analgesic, antitumor, or anti-inflammatory activities [5]. Many marine compounds are also immunomodulant [5], i.e., known to influence the immune system by either affecting the functions of immune cells or affecting antibody secretion to control infection and to maintain immune homeostasis [6]. For example, the marine dinoflagellate *Karenia brevis* (Davis) G. Hansen & Moestrup produces a number of bioactive compounds with therapeutic potential, including brevenal, which attenuates bronchoconstriction and increases tracheal mucosal velocity in sheep [7]. Brevenal was patented as a treatment for COPD, cystic fibrosis, and asthma [8].

Mucus is among the most intriguing substances secreted by animals, allowing them to adhere to the substratum [9] as well as acting as the first defence line against negative external forcing [10]. Indeed, mucosal properties evolved to facilitate the colonization of skin surfaces to specific microbial communities, allowing organisms to live in symbiosis with their hosts and contributing to organismal defences [10,11]. This biomechanism represents a physical and chemical barrier to harmful microorganisms as well as against external agents [12,13,14,15,16]. The study of epithelial mucus from the marine biota revealed the presence of bioactive and antimicrobial compounds [17], provided with potential immunomodulant activity which can activate the innate and acquired immune system [18]. Thus, the recognized mucus bioactivity might be a resource for human health [13], enhancing immune response against neoplastic cells and thus avoiding cancer diseases [19] and recognizing bacterial and viral pathogens that compromise health status [20].

Among invertebrates, the class Gastropoda Cuvier, 1797 (phylum Mollusca Cuvier, 1797) possesses epithelial gland cells through which they secrete mucus, accounting in this clade for two main functions: facilitating active locomotion, somehow lubricating the muscular foot, but mostly protecting and hydrating the mollusc epidermis under various stress conditions, which include desiccation but also infections or intrusions by viruses, bacteria, parasites, and predators [21]. Gastropod mucus typically contains mostly water, mucin-like molecules (protein-polysaccharide complex), electrolytes, epithelial and blood cells, and a wide range of molecules [22]. Its consistency, viscosity, and elasticity depend on the mucoprotein content and diversity associated with carbohydrates [22]. Indeed, the molecular weight of mucins or mucin-like glycoproteins ranges from 200 kDa to 200 MDa [23].

This study proposes a bioprospecting of the “mucus” immunomodulant activities of some marine gastropods. In particular, seven different species of macro-gastropods were selected from the Gulf of Naples (Mediterranean Sea) and eco-sustainably provided as by-catch product of commercial fishing activities. Firstly, the morphological taxonomic assignment of the seven species was confirmed by molecular tools. Then, the bioactivity of mucus on human cancer cell lines and monocytes was carried out. The most active mucus was finally targeted for further biological assessment focusing on the immunomodulatory activity capacity of the mucus through the expression of the key genes involved in specific stimulation of human monocytes.

## 2. Materials and Methods

### 2.1. Sampling

Molluscan samples were obtained from by-catch of commercial fishing activities held in the Gulf of Naples (central-western Mediterranean Sea) (Table 1). Shallow-water (≤50 m) samples were collected using trammel nets (1 m in height and ∼550 m in length; net consisting of an inner panel of 4.8 cm stretched mesh between two panels of 25 cm stretched mesh) fishing passively for 12–14 h, and deep-water (≥50 m) samples using bottom trawl nets (3 m high, 4 m wide mouth, 40 mm mesh size) towed for 3–4 h at ∼2.5 knots. A variable number of specimens (according to the species) was brought alive at Stazione Zoologica Anton Dohrn (SZN, Napoli, Italy) and subsequently kept for a week in the local aquarium facilities to let them acclimatize.

### 2.2. Morphological and Molecular Identification of Mollusca

Morphological identification of the sampled material was performed with magnifying lens and a Zeiss Axio Zoom.V16 microscope (Carl Zeiss, Oberkochen, Germany), paying attention to diagnostic shell characters and following the most recent guides on the molluscan biota of the Mediterranean Sea [24,25]. Then, soon after the mucus collection (see below), identifications were also confirmed through a DNA-barcoding approach. To do so, total genomic DNA was extracted from muscle samples using the DNeasy^®^ Blood & Tissue kit (Qiagen, Hilden, Germany), following the manufacturer’s protocol. A partial sequence of the *cox1* mitochondrial gene was amplified from one specimen per species using both the primers developed by Folmer et al. [26] (LCO-1490 (forward) 5′-GGTCAACAAATCATAAAGATATTGG-3′; HCO-2198 (reverse) 5′-TAAACTTCAGGGTGACCAAAAATCA-3′) and their degenerated version by Meyer [27] dgLCO-1490 (forward) 5′-GGTCAACAAATCATAAAGAYATYGG-3′; dgHCO-2198 (reverse) 5′-TAAACTTCAGGGTGACCAAARAAYCA-3′] (Table 1). The polymerase chain reactions (PCRs) were conducted in 25 μL volume reaction, containing 2.5 μL of Roche buffer (10×), 2.5 μL (2 mM) of dNTPack Mixture (Roche), 1 μL of each forward and reverse primers (10 µM), 0.25 μL (5 U/μL) of Roche Taq DNA polymerase, 1 μL of DNA (15 ng/μL), and sterilized distilled water up to 25 μL. Amplifications were performed with the following conditions: initial denaturation at 95 °C (5 min), followed by 39 cycles of denaturation at 95 °C (1 min), annealing at 42–45 °C (1 min) depending on the species (Table 1), extension at 72 °C (1 min), with a final extension at 72 °C (5 min). The successful PCR products were purified and Sanger sequenced through an Automated Capillary Electrophoresis Sequencer 3730 DNA Analyzer (Applied Biosystems, Waltham, MA, USA), using the BigDye^®^ Terminator v3.1 Cycle Sequencing Kit (Life Technologies, Carlsbad, CA, USA). Chromatograms for each sequence were then quality checked, assembled, and edited using Sequencher v.5.0.1 (GeneCodes, Ann Arbor, MI, USA). The identity of sequences obtained was finally checked through the Basic Local Alignment Search Tool (BLAST; www.ncbi.nih.gov/BLAST/, 31 May 2022) [28].

Sequenced samples were fixed in 99.9% ethanol and preserved in the collection of the Laboratory of Benthos-Napoli (SZN), under the voucher codes reported in Table 1. Updated taxonomy and nomenclature follow the World Register of Marine Species [29].

### 2.3. Mucus Collection

After the one week acclimatization period, three live specimens per species were selected among those who survived the sampling, isolated from the other samples, and placed in 150 × 15 mm plastic Petri dishes filled with 0.22 μM Millipore filtered natural sea-water. Then, once the muscular foot was exposed (about each 30 min), specimens were gently stung in their soft parts with laboratory tips to let them secrete mucus through mechanical stimulation. The mucus from the three specimens was finally collected time by time with a glass Pasteur pipette, transferred into a single 2 mL vial in ice, centrifuged at 10,000 rpm for 3 min before separating supernatant and filtering, and frozen [30].

### 2.4. Human Cell Lines Viability

The cytotoxic effect of the gastropods’ mucus was tested on lung adenocarcinoma cell line (A549) and human melanoma cell line (A2058) cells grown in DMEM F12, supplemented with 10% (v/v) fetal bovine serum (FBS) and 100 units mL^−1^ penicillin and 100 μg mL^−1^ streptomycin.

All cell lines were incubated in a 5% CO_2_ humidified chamber at 37 °C for growth. A2058 and A549 cells (20 × 10^3^ cells well^−1^) were seeded in a 96-well plate and kept overnight for attachment. The next day, the medium was replaced with fresh medium containing lyophilized mucus. The concentrations tested for the samples were 1, 10 and 100 μg mL^−1^. Cells were treated for 48 h, and adherent cells were then examined for viability. Cells were incubated with 10 μL (10 μg mL^−1^) of MTT (3-[4,5-methylthiazol-2yl]-2,5-diphenyl-tetrazoliumbromide). After 3 h of incubation, medium was removed, and the resultant formazan crystals were dissolved in isopropyl alcohol (100 μL). Absorbance intensity was measured with a microplate reader, at 570 nm. All experiments were performed in triplicate, and the number of viable cells was calculated as the ratio between mean absorbance of the sample and mean absorbance of untreated control cells and expressed as percentage viability.

### 2.5. Size and Morphology of Human Monocytes

Human monocytes (U937) (2 × 10^6^ cells) were seeded in 6-well plates. Samples were incubated with the seven gastropods’ mucus for 48 and 72 h. BD FACSVerse flow cytometry (BD Biosciences, Franklin Lake, NJ, USA) equipped with 488 nm argon laser and standard filter set was used to assess the changing of morphology and size of U937 after 48 and 72 h and to investigate the cytotoxic effect of *Bb*m in samples incubated for 24 h with three different concentrations of this mucus (1, 10 and 100 μg mL^−1^). The combination of Forward Scatter (FSC, commonly used as an indicative of cell size) and Side Scatter (SSC, used as an indicative of composition and/or complexity of the cells) was used to identify monocyte population. Acquisition was performed with BD FACSuite software. Data analysis and graphs were performed with FCS Express 6 Flow v 6.06.0025, DeNovo Software, Pasadena, CA, USA.

### 2.6. RNA Extraction and Real Time qPCR

To study the immunomodulatory activity of the *Bolinus brandaris*’ mucus (*Bb*m), U937 cells (human monocytes) were grown in RPMI medium, supplemented with 10% (v/v) FBS, 100 units mL^−1^ penicillin and 100 μg mL^−1^ streptomycin. U937 were incubated in a 5% CO_2_ humidified chamber at 37 °C for growth. U937 were seeded in 6-well plate (10 × 10^6^ cells well^−1^) and treated with 10 μg mL^−1^ of *Bb*m for gene expression analysis. RNA extraction was performed after 2 h of treatment; U937 cells were washed by adding cold PBS and rocking gently. Cells were lysed by adding 1 mL of Trisure Reagent. RNA was isolated according to the manufacturer’s protocol. RNA concentration and purity was assessed using the nanophotomer NanodroP (Euroclone). RNA (200 ng) was subjected to reverse transcription reaction using the RT2 first strand kit (Qiagen) according to the manufacturer’s instructions. Real-Time qPCR was performed in triplicate using the RT^2^ Profiler PCR Array kit (RT² Profiler™ PCR Array Human Innate & Adaptive Immune Responses, Qiagen) to analyse the expression of inflammation cell signaling genes in the U937 cells. Plates were run on a ViiA7 (Applied Biosystems 384 well blocks), Standard Fast PCR cycling protocol with 10 μL reaction volumes. Cycling conditions used were 1 cycle initiation at 95.0 °C for 10 min followed by amplification for 40 cycles at 95.0 °C for 15 s and 60.0 °C for 1 min. Amplification data were collected with ViiA 7 RUO Software (Applied Biosystems, Waltham, MA, USA). Ct values were analysed with PCR array data analysis online software (http://pcrdataanalysis.sabiosciences.com/pcr/arrayanalysis.php, accessed on 30 April 2022, Qiagen).

### 2.7. ELISA Assay

In order to assess the effect of *Bb*m on the release of IL-6, U937 cells (2 × 10^6^ cells) were seeded in 6-well plates (TPP Techno Plastic Products AG, Trasadingen, Switzerland) and kept overnight for attachment. U937 cells were treated with 10 μg mL^−1^ of *Bb*m and, after 24 h, media were collected from control (no *Bb*m) and treated cells. After incubation, cell media were collected and used to evaluate the release of cytokines, by sandwich ELISA detection, using Human IL-6 Standard ABTS ELISA Development Kit (cat. No. 900-K16, PeproTech, London, UK), according to manufacturer’s protocol. The absorbance was measured at 405 nm (with wavelength correction set at 650 nm) using Microplate Reader: Infinite^®^ M1000 PRO (Ex: 320 nm, Em: 420 nm, TECAN, Männedorf, Switzerland). IL-6 levels were expressed in ng mL^−1^ of medium (using IL-6 standard curve).

### 2.8. Immunoarray Analysis

The regulation of the proteins involved in the inflammation process was investigated through an antibody array performed using RayBiotech^®^ C-Series Human Inflammation Array C3 (code: AAH-INF-3, RayBiotech, Peachtree Corners, GA, USA). For this aim, U937 cells (2 × 10^6^ cells^−1^) were seeded in 6-well plates (TPP Techno Plastic Products AG, Trasadingen, Switzerland). For the detection of proteins, cells were treated for 24 h at a concentration of *Bb*m of 10 μg mL^−1^. After incubation, cell medium was collected from control and treated cells. Protein concentration and purity were assessed using the NanoDrop 1000 Spectrophotometer (Thermo Fisher Scientific, Waltham, MA, USA) and 1 mL of sample was used to perform the antibody array, according to the manufacturer’s protocol. Blots were analyzed using ImageLab software (Bio-Rad, Hercules, CA, USA) and results are shown in terms of relative expression.

### 2.9. Statistical Analyses

All experiments were performed in triplicate. GraphPad Prism 8.0 was used for statistical analysis. Student’s *t*-test was used to compare the differences between two groups. Statistical differences of multiple groups were determined by two-way analysis of variance (ANOVA) followed by Tukey’s or Sidak’s post hoc test. The mean ± standard deviation of the mean (SD) was used to express the data. A *p*-value of less than 0.05 was considered statistically significant and the statistical differences were represented as follows: * *p* ≤ 0.05, ** *p* ≤ 0.01, *** *p* ≤ 0.001 and **** *p* ≤ 0.0001.

## 3. Results

### 3.1. Species Identification

Over the seven species, six of them belonged to the subclass Caenogastropoda Cox, 1960, namely *Aptyxis syracusana* (Buccinoidea: Fasciolariidae), *Bolinus brandaris* (Muricoidea: Muricidae), *Euthria cornea* (Buccinoidea: Tudiclidae), *Galeodea echinophora* (Tonnoidea: Cassidae), *Monoplex corrugatus* (Tonnoidea: Cymatiidae), and *Naticarius stercusmuscarum* (Naticoidea: Naticidae), whereas one belonged to the subclass Vetigastropoda Salvini-Plawen, 1980, namely *Bolma rugosa* (Trochoidea: Turbinidae) (Table 1). The morphological taxonomic assignment was confirmed by DNA barcoding. *Aptyxis siracusana* showed a 100% similarity with the single mitochondrial *cytochrome c oxidase subunit 1* gene locus (*cox1*) sequence of this species available in GenBank (KT753968) and based on a sample from Tunisia (Mediterranean Sea) [31]. *Bolinus brandaris* showed high (99.12–99.24%) similarity with the two *cox1* sequences of this species available in GenBank (DQ280020 and EU827194) and based on samples from unspecified localities [32,33]. *Euthria cornea* showed a 98.89% similarity with the single *cox1* sequence of this species available in GenBank (MW077006) and based on a sample from Corse (France, Mediterranean Sea) [34]. *Galeodea echinophora* showed a 100% similarity with the two *cox1* sequences of this species available in GenBank (KP716635 and MH581337) and based on samples from Valencia (Spain, Mediterranean Sea) and the Mediterranean Sea [35,36]. *Bolma rugosa* showed high (98.94–99.69%) similarity with the two *cox1* sequences of this species available in GenBank (AM049372 and KT207824) and based on samples from Gulf of Naples (Italy, Mediterranean Sea) and Chafarinas Islands (Spain, Mediterranean Sea) [37,38]. *Naticarius stercusmuscarum* showed a 100% similarity with the single *cox1* sequence of this species available in GenBank (EU332644) and based on a sample from Giglio Island (Italy, Mediterranean Sea) [39]. Within all these BLASTn queries, other non-conspecific taxa showed similarities ≤93.77%, thus well over the barcoding gap commonly accepted in molluscs (~3%) (e.g., [40]). Therefore, molecular results confirmed the morphological identifications for six species. On the other hand, no *cox1* sequences were available for *M. corrugatus*, and thus its partial *cox1* sequence was first deposited in GenBank based on the present sample. The BLASTn query also showed a maximum similarity (94.73%) with a sequence (MH581346) of the congeneric species *Monoplex krebsii* (Mörch, 1877) deposited by Strong et al. [36], thus excluding contamination, or eventually misidentification.

### 3.2. Human Cell Viability Assessment

**Figure 1:**
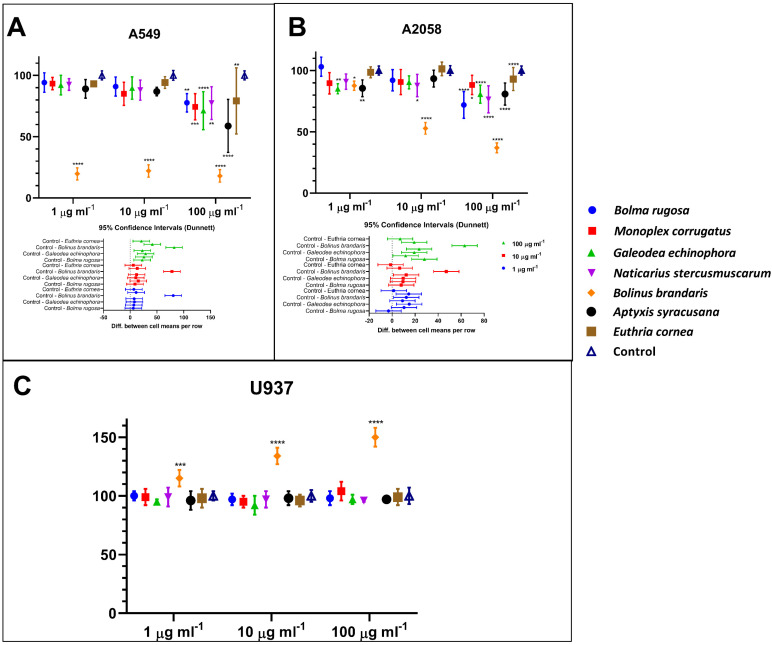
Percentage of human cell lines treated during 48 h with 1, 10 or 100 µg mL^−1^ of lyophilized mucus extracted or isolated from marine gastropods. Panels (**A**–**C**) represent results obtained by MTT assay on A549, A2058, and normal monocyte cell lines (U937) treated with lyophilized mucus. Statistical significance is calculated through Dunnett test and is indicated by **** (*p* ≤ 0.0001), *** (*p* ≤ 0.0005), ** (*p* ≤ 0.005), and * (*p* ≤ 0.05). Values are expressed as mean ± standard deviation.

Mucus from the different species did not display the same bioactivity capacity on the cancer cell lines (Figure 1A,B). Interestingly, none of the mucus tested is cytotoxic for normal human cells U937 (Figure 1C). By contrast, the *Bolinus brandaris* mucus (*Bb*m) enhanced the metabolic capacity of normal cells, indicating a potential immunostimulant effect (Figure 1C). Interestingly, *Bb*m mucus displayed significant cytotoxic effect (*p* ≤ 0.0001) on the two cancer cell lines, with an IC50 ≤ 1 µg mL^−1^ and ~10 µg mL^−1^ for the A549 and A2058 cell lines, respectively. The other mucus did not exert significant cytotoxic activity on the human cancer cell lines, except for a few exceptions at the highest mucus concentration (Figure 1A,B).

### 3.3. Morphological Changes of Monocytes

**Figure 2:**
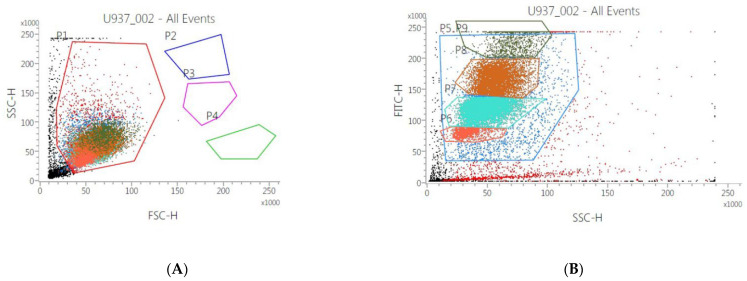
Flow cytometry analysis U937 incubated with *Bolinus brandaris* mucus (*Bb*m). The combination of FSC (proportional to cell-surface area or size) and SSC (proportional to morphology, cell granularity, or internal complexity) was used to discriminate human monocyte population P1 (in red) in all the dot plot. In (**A**), it is represented the control (untreated cells). (**B**) represented cells incubated with 100 µg mL^−1^ of *Bb*m respectively. P2–P9 indicate the changes in morphology and size of the monocyte population when in contact with the highest *Bb*m concentration.

In order to assess the effect of the mucus on human monocytes, U937 cell samples were incubated for 48 and 72 h with gastropod mucus from the seven species indicated in Table 1 and analyzed by flow cytometer. The results demonstrated that only cells incubated with *Bolinus brandaris* exhibit an effect on size (FSC-H: Figure 2A) and morphology (SSC-H: Figure 2B), with a significant differentiation in terms of 9 cells’ subpopulation. These results are more significant at 48 h than those at 72 h of incubation. On the other hand, the mucus from the other gastropod species did not show any difference either in size or in morphology both at 48 and 72 h. Furthermore, in all samples, cells concentration increased about 40% after 72 h of incubation (with respect to 48 h). Finally, to assess the cytotoxic effect of *Bb*m, samples were incubated with different concentrations of mucus (1, 10 and 100 μg mL^−1^). Results showed that only the highest concentration induced both a strong morphological effect and a decrease in cell concentration of about 78% compared to untreated cells or to cells treated with low concentration of *Bb*m.

### 3.4. Bbm-Induced Gene Expression in U937 Cells

**Figure 3:**
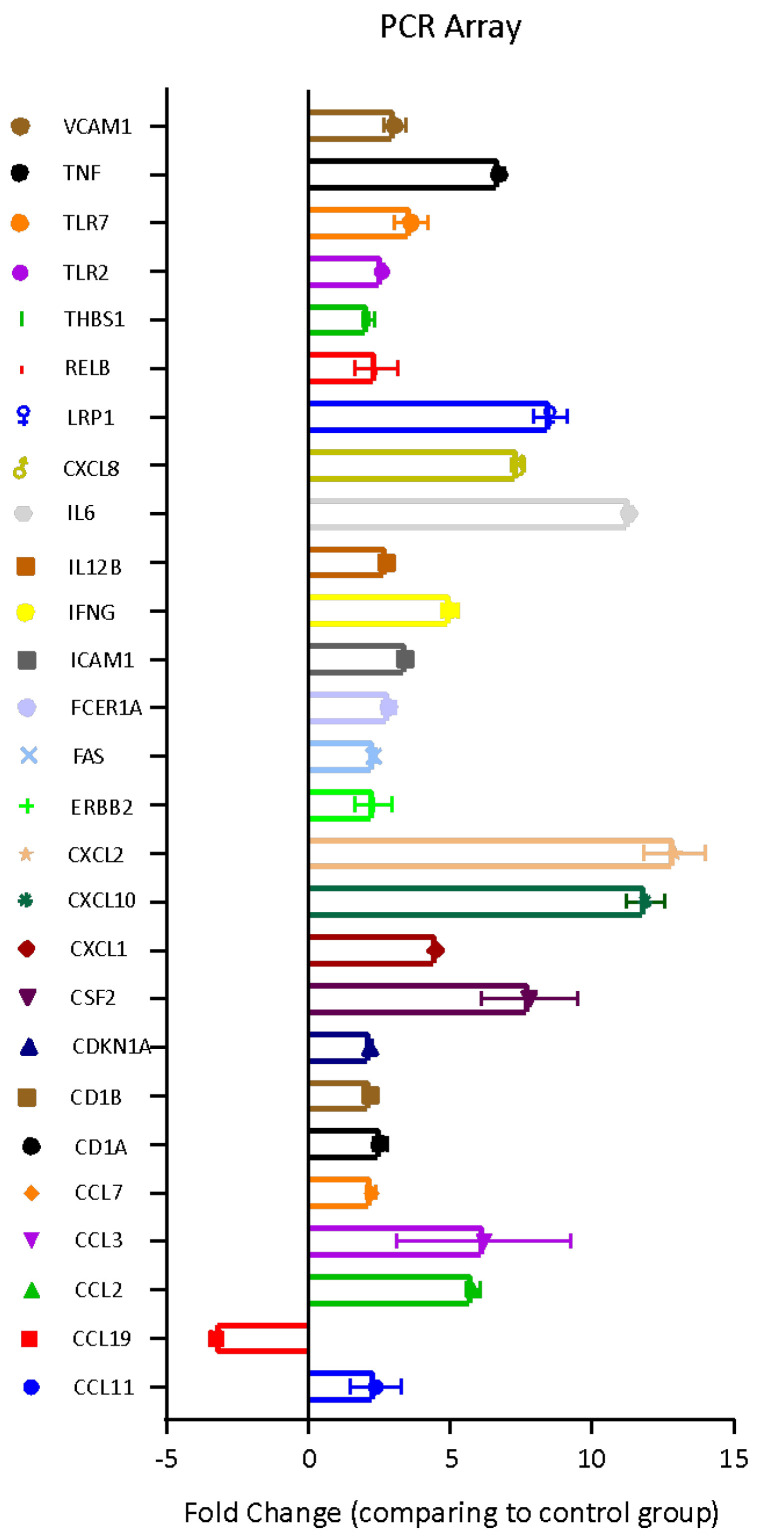
Fold regulation (with respect to the untreated U937 cells) of 27 genes implicated in inflammatory response in U937 cells treated with *Bb*m.

The molecular effects induced by the *Bolinus brandaris* mucus on U937 cells were investigated through the analysis of the expression level of 84 genes involved in the Human Innate and Adaptive Immune Responses. Twenty-six genes were up-regulated whereas one gene displayed a significant down-regulation (Figure 3).

*Bb*m did also affect the pro-inflammatory and immunomodulatory factors, such as Interleukins 12 beta and 6 (IL-12B and IL-6), Low density lipoprotein receptor-related protein 1 (LRP1), V-rel reticuloendotheliosis viral oncogene homolog B (RELB), Thrombospondin 1 (THBS1), Toll Like Receptors 2 and 7 (TLRs 2 and 7), tumor necrosis factor (TNF), which were all up-regulated (Figure 3). Yet, the Vascular cell adhesion molecule 1 (VCAM1) was overexpressed by *Bb*m indicating the involvement of vasodilator factors for the recruitment of immune system cells in the immunomodulatory activity.

### 3.5. Bbm-Induced Interleukin IL-6 Release in U937 Cells

**Figure 4:**
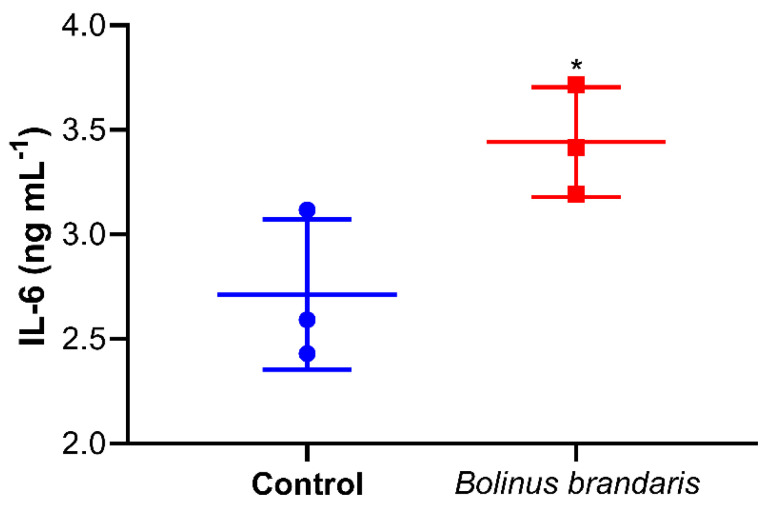
The effect of *Bb*m on serum-released interleukin 6 (IL-6) in human monocytic cells (U937). Values are expressed as average of IL-6 concentration (ng mL^−1^) determined by ELISA in culture medium of cells treated with 10 μg mL^−1^ of *Bb*m and untreated cells (Control) for 24 h. n = 3; * *p* ≤ 0.05 (Student’s *t*-test analysis).

Since the IL-6 gene was one of the most overexpressed genes of U937 treated with *Bb*m, the production of IL-6 by U937 cells stimulated was estimated displaying a concentration of 3.5 ng mL^−1^ vs. 2.6 ng mL^−1^ in the control (Figure 4).

### 3.6. Bbm-Induced Inflammation Mediators Release in U937 Cells

**Figure 5:**
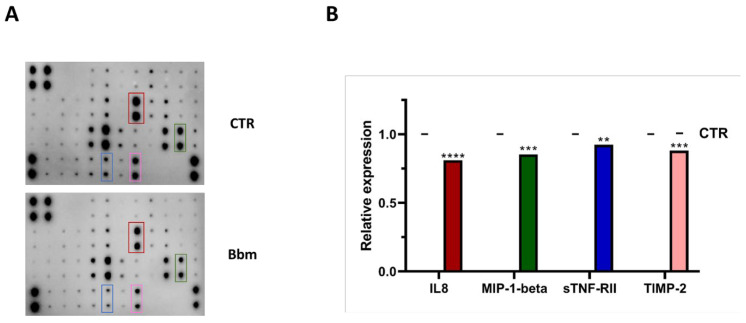
(**A**) Human inflammation array analysis of the conditional medium from U937 cells treated with 10 μg mL^−1^ of *Bolinus brandaris* mucus (*Bb*m). (**B**) Relative expression levels of the four inflammation cytokines, IL-8, MIP-1β, sTNF-RII and TIMP-2. Statistical significance is calculated through Sidak’s test and is indicated by **** (*p* ≤ 0.0001), *** (*p* ≤ 0.0005), ** (*p* ≤ 0.005). Values are expressed as mean ± standard deviation.

The inflammatory mediators, IL-8, MIP-1β, sTNF-RII and TIMP-2, were significantly lowered in the medium of the U937 cell line treated with *Bb*m compared to the untreated condition (Figure 5A,B), with IL-8 and MIP-1β release down-expressed by 35 and 25%, respectively.

## 4. Discussion

In the present study, the mucus was isolated and lyophilized from seven different gastropod species. In general, the mucus antiproliferative activity on lung adenocarcinoma (A549 cell line) and melanoma (A2058) depends on the concentration, but not all mucus displays significant bioactivity in inducing cell death in cancer cell lines. This probably depends on the different bioactive molecules contained in the mucus from the different species, as highlighted in the literature [4] and refs therein. The most bioactive mucus is that obtained from *B. brandaris*, with a significant antiproliferative effect already at the minimum concentration tested (1 μg mL^−1^), together with an increase in metabolically active cells in the human monocytes U937 line. This species has been reported since antiquity to have different human health benefits, depending on the parts of the animal considered and was prescribed by clinical homeopaths as remedy [4] and refs therein. Our results demonstrate that *Bb*m leads to an immunomodulation of innate immunity for the control of the adaptive immune response at various levels avoiding inflammatory storm, as revealed for instance by the overexpression of Toll like receptors (TLRs). In particular, TLR2 and TLR7 are targeted by *Bb*m, indicating cell stimulation for a more efficient recognition of pathogens or part of them, such as lipoproteins and other microbial cell wall components. These signals lead to the activation of innate immunity [41]. The activation of TLRs then induce an increase in gene expression of the transcription factors that regulate inflammatory and immune responses, such as chemokines CCLs. Chemokines, including CCL2, CCL3, CCL7 and CCL11, are up-regulated. This gene family is regulated at transcriptional level during inflammation and encodes for small, structurally related, chemoattractant molecules. Chemokines are also involved in the recruitment of the inflammatory competent cells to target tissues (e.g., monocytes, macrophages, eosinophils and basophils), regulating cell trafficking. Besides chemotaxis, chemokines are also involved in the regulation of T cell differentiation, apoptosis, cell cycle, angiogenesis, and metastatic processes [42]. Furthermore, chemokines can control the generation of soluble inflammatory products such as free radicals and cytokines [43]. The other chemokine, CXCL8, activated by *Bb*m, encodes for a key mediator of inflammation (IL-8). This protein is a chemotactic factor that guides the neutrophils to the site of infection. This chemokine acts, together with CXCL1, CXCL2 and CXCL10 (also up-regulated), as a potent angiogenic factor, recruiting endothelial cells [44]. Other angiogenic molecules, such as the glycoprotein THBS1, is up-regulated by *Bb*m; this molecule mediates cell-to-cell and cell-to-matrix interactions. The gene encoding for a cytokine that can induce cell death in no-self cells and cancer cells [45] is also up-regulated, together with LRP1, with which the former interacts, activating apoptotic cell death [46]. The interaction with IL-6 can trigger the activation of netosis [47]. The *Bb*m-induced activation of genes related to the host defence in U937, such as CD1A, CD1B, and IFNG, is essential in the inflammatory cascade triggered by the innate immunity before the activation of the secondary immunity. This response causes the release of secondary mediators responsible for the plethora of symptoms associated with inflammatory diseases. Among all involved mediators, IL-6 has a central role in these mechanisms, being a cytokine that acts in inflammation and maturation of B cells. IL-6 is released at the sites of acute and chronic inflammation, where it induces a transcriptional inflammatory response through the interleukin 6 receptor alpha. The up-regulation of the IL-6 gene, and the increase in Il-6 cytokine synthesis, confirms the inflammatory response induced by *Bb*m, which starts the innate immunity response by triggering signalling for the lymphocyte recruitment, also demonstrated by the size changes observed by FACS analysis of the naive monocytes U937.

Indeed, MIP-1β produced by macrophages and monocytes after proinflammatory injuries [48] is significantly down expressed. It plays a crucial role in immune responses towards infection and inflammation. It also induces the synthesis and release of other pro-inflammatory cytokines such as interleukin 1 (IL-1), IL-6 and TNF-α from fibroblasts and macrophages.

*Bb*m induces a slight decrease in sTNF-RII release in U937. Interestingly, when the soluble Tumor necrosis factor receptor II (sTNF-RII) increases in blood and plasma serum, it seems to be associated with a higher risk in cancer diseases, and it has been also associated with overall pathogenicity and mortality [49]. This sTNF-RII decrease in U937 might be paralleled with the significant antiproliferative activity of *Bb*m on the cancer cell lines. *Bb*m also induces a decrease of Tissue Inhibitor of Metalloproteinase-2 (TIMP-2) expression indicating an involvement of *Bb*m in the cell cycle promotion in human monocytes, as also demonstrated by the FACS analysis. TIMP-2 expression is induced by cytokines and chemokines and proliferation (βFGF and EGF) and differentiation (retinoic acid and NGF) factors [50]. The role of TIMP-2 in the innate immunity is represented by structural changes influencing leukocyte transmigration from the capillaries to areas of injury in the renal tubule [50], changes in endothelial permeability and modulation of the inflammatory response [51], apoptosis (cell death) [52], and finally loss of cell–cell adhesion and sloughing of tubular epithelial cells.

## 5. Conclusions

Blue biotechnology is considered an opportunity for the sustainable development of new products through the exploration and exploitation of marine organisms. Among marine invertebrate resource and diversity, gastropods are known to produce and secrete mucus, often displaying bioactive properties. The bioprospecting conducted in our study reports that from seven species inhabiting the Gulf of Naples, *Bolinus brandaris* secretes mucus with human health potential and induces a modulation of innate immunity for the control of the adaptive immune response. This might be of great biotechnological interest, as immune system dysfunction is a very important issue in human health protection.

## Figures and Tables

**Table 1:** Taxa analyzed in the present study, with sampling depth range (in meters), coordinates (latitude and longitude), primers used (P), temperature of annealing (in degrees) (T), base pairs amplified (bp), voucher code (SZN Laboratory of Benthos-Napoli: SZN_B_), and GenBank accession number.

Taxon	Depth Range	Coordinates	P	T	bp	Voucher	GenBank
Caenogastropoda							
*Aptyxis syracusana* (Linnaeus, 1758)	20–30 m	40.8181 N, 14.1195 E	dgLCO-1490 dgHCO-2198	45	701	1387ML49E	ON926803
*Bolinus brandaris* (Linnaeus, 1758)	75–150 m	40.8067 N, 14.1401 E	dgLCO-1490 dgHCO-2198	45	683	1183ML113B	ON926804
*Euthria cornea* (Linnaeus, 1758)	20 m	40.8181 N, 14.1195 E	dgLCO-1490 dgHCO-2198	42	697	1168ML10B	ON934996
*Galeodea echinophora* (Linnaeus, 1758)	100–350 m	40.7503 N, 14.1128 E	LCO-1490 HCO-2198	45	682	248ML107D	ON926805
*Monoplex corrugatus* (Lamarck, 1816)	50–100 m	40.8067 N, 14.1401 E	LCO-1490 HCO-2198	45	608	464ML19A	ON930589
*Naticarius stercusmuscarum* (Gmelin, 1791)	100–250 m	40.7503 N, 14.1128 E	dgLCO-1490 dgHCO-2198	42	684	460ML18D	ON926806
Vetigastropoda							
*Bolma rugosa* (Linnaeus, 1767)	20–30 m	40.8181 N, 14.1195 E	dgLCO-1490 dgHCO-2198	42	663	2894ML197A	ON926807

## Data Availability

Not applicable.
